# Upregulation of sICAM-1 and sVCAM-1 Levels in the Cerebrospinal Fluid of Patients with Schizophrenia Spectrum Disorders

**DOI:** 10.3390/diagnostics11071134

**Published:** 2021-06-22

**Authors:** Sophie Meixensberger, Hanna Kuzior, Bernd L. Fiebich, Patrick Süß, Kimon Runge, Benjamin Berger, Kathrin Nickel, Dominik Denzel, Miriam A. Schiele, Maike Michel, Simon Maier, Karl Bechter, Katharina Domschke, Ludger Tebartz van Elst, Dominique Endres

**Affiliations:** 1 Section for Experimental Neuropsychiatry, Department of Psychiatry and Psychotherapy, Medical Center—University of Freiburg, Faculty of Medicine, University of Freiburg, 79104 Freiburg, Germany; kimon.runge@uniklinik-freiburg.de (K.R.); kathrin.nickel@uniklinik-freiburg.de (K.N.); dominik.denzel@uniklinik-freiburg.de (D.D.); simon.maier@uniklinik-freiburg.de (S.M.); 2 Department of Psychiatry and Psychotherapy, Medical Center, Faculty of Medicine, University of Freiburg, 79104 Freiburg, Germany; bernd.fiebich@uniklinik-freiburg.de (B.L.F.); miriam.schiele@uniklinik-freiburg.de (M.A.S.); maike.michel@uniklinik-freiburg.de (M.M.); katharina.domschke@uniklinik-freiburg.de (K.D.); 3 Department of Radiology, Medical Center, Faculty of Medicine, University of Freiburg, 79106 Freiburg, Germany; hanna.kuzior@uniklinik-freiburg.de; 4 Department of Molecular Neurology, University Hospital Erlangen, 91054 Erlangen, Germany; Patrick.Suess@uk-erlangen.de; 5 Clinic of Neurology and Neurophysiology, Medical Center, Faculty of Medicine, University of Freiburg, 79110 Freiburg, Germany; benjamin.berger@uniklinik-freiburg.de; 6 Department of Psychiatry and Psychotherapy 2, Ulm University, Bezirkskrankenhaus Günzburg, 89312 Günzburg, Germany; karl.bechter@bkh-guenzburg.de; 7 Center for Basics in Neuromodulation, Faculty of Medicine, University of Freiburg, 79106 Freiburg, Germany

**Keywords:** ICAM-1, VCAM-1, schizophrenia, depression, neuroinflammation, blood-brain barrier, cerebrospinal fluid

## Abstract

Immunological explanatory approaches are becoming increasingly important in schizophrenia research. In this context, the function of the blood-brain barrier (BBB) and the blood-cerebrospinal fluid (CSF) barrier (BCSFB) plays an essential role. Different adhesion molecules, such as intercellular adhesion molecule-1 (ICAM-1) and vascular cell adhesion molecule-1 (VCAM-1), are key elements in sustaining the integrity of the BBB and BCSFB. The objectives of this study were to (1) compare the levels of different cell adhesion molecules in the CSF of patients with schizophrenia spectrum disorders to those of patients with unipolar depression and (2) analyze their association with the established markers of the BBB/BCSFB function (CSF total protein and albumin quotient (AQ)). Therefore, a total of 40 patients with schizophrenia spectrum disorder and 39 age- and sex-matched control patients with unipolar depression were analyzed. The levels of soluble ICAM-1 (s-ICAM-1), soluble VCAM-1 (s-VCAM-1), and plasminogen activator inhibitor 1 (PAI-1) in the CSF were measured using a magnetic bead multiplexing immunoassay. The levels of sICAM-1 (*p* < 0.001), sVCAM-1 (*p* < 0.001), and PAI-1 (*p* < 0.001) in the CSF were significantly higher in patients with schizophrenia spectrum disorder than in patients with unipolar depression. In addition, a significant correlation of sVCAM-1 levels with total protein concentrations (r = 0.454, *p* = 0.003) and AQ levels (r = 0.512, *p* = 0.001) in patients with schizophrenia spectrum disorders was observed. The results revealed that sICAM-1 and sVCAM-1 levels in the CSF were higher in patients with schizophrenia spectrum disorder than in those with depression. These circulating signaling molecules may indicate endothelial dysfunction causing impaired BBB/BCSFB function in patients with schizophrenia spectrum disorders. Consistent with this view, a highly significant correlation of sVCAM-1 with CSF protein and AQs was detected. Upregulation of these cell adhesion molecules might be indicative of a proinflammatory immune response underlying the BBB/BCSFB disturbance in a subgroup of patients with schizophrenia spectrum disorders. The significance of the study is limited by its retrospective research design and by the absence of a healthy control group. The assay used was not previously established for the measurement of CSF. Further translational and controlled studies of the role of different cell adhesion molecules in schizophrenia are needed.

## 1. Introduction

Immunological explanatory approaches are becoming increasingly important in schizophrenia research [1]. Schizophrenia spectrum disorders have been interpreted by several authors as complex neuropsychiatric disorders involving an activated inflammatory response leading to mild neuroinflammation [2,3,4,5]. In this context, the blood-brain barrier (BBB) and the blood-cerebrospinal fluid (CSF) barrier (BCSFB) play a central role [6], and a number of clinical studies have shown alterations in biomarkers associated with the BBB/BCSFB [7,8,9,10,11]. The central nervous system (CNS) is surrounded by the dynamic and metabolically active CSF and is separated from the peripheral circulation by several barriers. The most prominent are the BBB and the BCSFB [12,13,14,15]. The BBB/BCSFB form the primary interface that exerts key functions in brain homeostasis and immune protection [9,12]. One of the most notable components responsible for barrier integrity is the brain capillary endothelial cells that sustain a paracellular pathway with highly selective permeability mediated by selective transport vesicles and tight junctions [6,16,17]. In this cerebral microvascular endothelium, different intercellular immunoglobulin (Ig)-like transmembrane glycoproteins, particularly intercellular adhesion molecule-1 (ICAM-1) and vascular cell adhesion molecule-1 (VCAM-1), are expressed under chronic inflammatory conditions [3,18]. Endothelial cells are not only a passive barrier but also immunologically active themselves. For example, they can produce chemokines [19], and endothelial VCAM-1 is associated with age- and inflammation-induced microglia activation, impaired neurogenesis, and cognitive deficits. These changes are diminished by the antagonization of VCAM-1 and can occur even without a disturbance of the BBB/BCSFB parameters or infiltration of immune cells [20]. Previous studies comparing patients with schizophrenia spectrum disorder and controls revealed contradictory findings on soluble ICAM-1 (sICAM-1) levels when obtained using different methods and samples [3]. These samples include serum, CSF, and postmortem CNS tissues, as summarized in Table 1. Two previous studies examining CSF samples found an association of sICAM-1-levels and BCSFB markers (total CSF protein and albumin quotient) and with clinical parameters (negative symptoms and duration of disease) [21,22].

The objective of this study was to conduct the first controlled CSF study to investigate cell adhesion molecules in patients with schizophrenia spectrum disorders and in a psychiatric control group. More specifically, we (1) compared the levels of different cell adhesion molecules in the CSF of patients with schizophrenia spectrum disorders to those of patients with unipolar depression and (2) analyzed the association of these cell adhesion molecules with the established CSF markers of BBB/BCSFB function (i.e., total CSF protein and AQ).

## 2. Patients and Methods

This study was part of a larger retrospective project approved by the local ethics committee (Faculty of Medicine, University of Freiburg, ethical vote no. 396/18). Lumbar punctures were performed after a careful gathering of information and after obtaining written informed consent as part of a clinical routine to rule out organic causes of psychiatric symptoms.

### 2.1. Study Sample

A total of 40 patients over 18 years old diagnosed with schizophrenia spectrum disorder and 39 patients diagnosed with unipolar depression were included in this study (for clinical and demographic details, see Table 2 and Table 3). Based on the predominant clinical syndrome, patients were classified according to the criteria set by the International Statistical Classification of Diseases and Related Health Problems, 10th revision (ICD-10). In the schizophrenia cohort, 13 patients went through their first episode and 27 suffered from a chronic or recurrent manifestation, with chronic being defined as a period of more than two years. In the depression cohort, 12 patients suffered from their first episode and 27 were in a chronic or recurrent stage. All 39 patients in the depression cohort were diagnosed with severe depressive episodes. Patients with acute infections, autoimmune diseases with well-known brain involvement, and neurodegenerative diseases were excluded.

### 2.2. Cerebrospinal Fluid Analysis and Instrumental Diagnostics

The routine CSF analysis included the determination of white blood cell (WBC) count, protein concentration, AQ, immunoglobulin (Ig)G index, and oligoclonal bands (OCBs) according to an established methodology (c.f. [7,8]). The measurements were carried out in the CSF laboratory of the University Hospital Freiburg (https://www.uniklinik-freiburg.de/neurologie/klinik/diagnostische-einrichtungen/liquor-labor.html, accessed on 12 June 2021). Electroencephalography (EEG) and cerebral magnetic resonance imaging (MRI) were offered to all patients as part of the routine clinical work-up.

### 2.3. Measurement of Cell Adhesion Markers

The measurements were performed retrospectively from CSF samples frozen at −80 °C. The adhesion molecules were quantified using a magnetic bead-based multiplex immunoassay using a Human Adhesion Magnetic 6-Plex Panel (ThermoFisher, Waltham, MA, USA). A MAGPIX^®^ machine (ThermoFisher, Waltham, MA, USA) was used to read and analyze the assay. The panel utilized to investigate sICAM-1, soluble VCAM-1 (sVCAM-1), plasminogen activator inhibitor 1 (PAI-1), P-selectin, E-selectin, and platelet endothelial cell adhesion molecule-1 (PECAM) was used in accordance with the manufacturer’s specifications. That was with the exception of the specification to use undiluted CSF samples, as this panel was originally not established for CSF analysis. The reported values were corrected for the different dilution. To determine whether the calculated concentrations of the individual adhesion molecules were reliable, we investigated the mean fluorescent intensity after deducting the blank value, which is known as the net median fluorescence intensity (NetMFI). We also investigated the number of magnetic beads measured per analyte per well (bead count; cf. [25]). In this study, all values with a NetMFI below the lowest standard of the standard curve of the respective cell adhesion molecule and all wells with a bead count below 20 were excluded (cf. [25]). Only the samples that were measurable (and therefore not below the detection level) for >50% of the analytes were analyzed. The adhesion molecule concentrations below the detection level were set to zero.

### 2.4. Data Handling and Statistical Analyses

Data were analyzed using the Statistical Package for the Social Sciences (SPSS), version 24 (IBM Corp., Armonk, NY, USA). Group comparisons for categorical variables were conducted using the Pearson’s chi-squared test or Fisher’s exact test in case any cell of the contingency table was below 5. For group comparisons of continuous variables, nonparametric Mann-Whitney U tests were used, since normal distribution according to the Shapiro–Wilk test could only be assumed for IgG index in schizophrenia spectrum disorders, PAI-1 concentrations in the depression group, as well as age, PAI-1, and sVCAM-1 concentrations in the schizoaffective subgroup. In a secondary analysis, the influence of medication on cell adhesion molecule concentrations was investigated by performing an analysis of covariance (ANCOVA) with different groups of medication (antidepressants, antipsychotics, anticonvulsants, and lithium) as covariates. Spearman’s rank correlation between cell adhesion molecules (sICAM-1, sVCAM-1, and PAI-1) and CSF basic parameters (WBC count, protein concentration, AQ, and IgG index) or clinical features (number of suicide attempts or earlier inpatient stays) was separately performed for each group (schizophrenia spectrum disorder and unipolar depression). A *p*-value of <0.05 was set to indicate statistical significance. No correction for multiple testing was performed, given that an exploratory approach was implemented in this study.

## 3. Results

### 3.1. Sociodemographic Data

The sociodemographic data are summarized in Table 2 and Table 3. The schizophrenia spectrum and depressive patient groups were matched for age (z = −0.388, *p* = 0.698) and sex (Chi^2^ = 0.141, *p* = 0.707). A difference in the level of education could be observed (*p* = 0.013).

### 3.2. Cell Adhesion Molecules in the Cerebrospinal Fluid

The cell adhesion molecules sICAM-1, sVCAM-1, and PAI-1 in the CSF were successfully measured. The other parameters could not be measured sufficiently. The levels of sICAM-1 (*p* < 0.001), sVCAM-1 (*p* < 0.001), and PAI-1 (*p* < 0.001) in the CSF were significantly higher in the patients with schizophrenia spectrum disorder than in those with unipolar depression (Table 4). Subgroup analyses between patients with schizoaffective disorder (*n* = 11) and the other patients from the schizophrenia spectrum disorder group (*n* = 29) had similar mean ages (z = −1.061, *p* = 0.289). Both groups did not differ in the concentrations of sICAM-1 (z = −0.939, *p* = 0.348), sVCAM-1 (z = −1.530, *p* = 0.126), and PAI-1 (z = −1.636, *p* = 0.102). The sICAM-1, sVCAM-1, and PAI-1 levels in patients with first-episode schizophrenia spectrum disorder or depression did not significantly differ from those in patients with a chronic/recurrent state of either disease (data not shown in detail).

In a secondary analysis correcting for the influence of psychotropic drugs, the group effects of all cell adhesion molecules remained significant. No significant effects of the main medication groups (antidepressants, antipsychotics, anticonvulsants, lithium) or group interactions were observed.

### 3.3. Basic Cerebrospinal Fluid Findings and Instrumental Diagnostics

The routine findings for CSF diagnostics are presented in Table 5. Overall, no significant differences in WBC counts, protein concentration, AQs, IgG indices, or rate of OCBs were observed between the schizophrenia and depression groups.

In total, 25/40 patients (63%) of the schizophrenia-spectrum disorder group had MRI alterations, 24/36 patients (67%; MRIs not available in three patients) of the depressive disorder group hat MRI alterations (Chi^2^ = 0.278, *p* = 0.598). White matter lesions/cerebral microangiopathy were most common (in 30% of patients with schizophrenia spectrum disorders and in 42% of patients with depressive disorders). EEG alterations were detected more frequent in patients with schizophrenia-spectrum disorders (in 10/40 patients; 25%) compared to patients with depressive disorders (in 2/39 patients; 5%; Chi^2^ = 6.053, *p* = 0.014). In the schizophrenia-spectrum disorder group, nine patients (23%) had intermittent generalized slow activity and one patient had intermittent focal slow activity (3%). In the patients with depressive disorders, one patient (3%) had continuous slow activity and one patient had epileptiform discharges (3%).

### 3.4. Correlation Analyses

**Figure 1:**
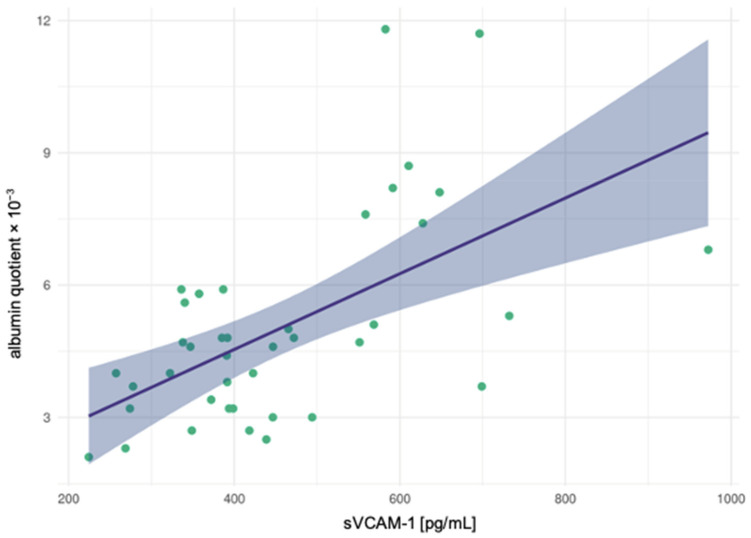
The albumin quotient significantly correlated with the vascular cell adhesion molecule-1 (sVCAM-1) in patients with schizophrenia spectrum disorders.

In the schizophrenia spectrum disorder cohort, the sVCAM-1 levels correlated significantly with the CSF total protein concentration (r = 0.454, *p* = 0.003) and the AQ (r = 0.512, *p* = 0.001; see Figure 1). By contrast, the levels of the cell adhesion molecules were not significantly correlated with clinical features (suicide attempts and the number of earlier inpatient stays). In the unipolar depression cohort, no significant correlations of sICAM-1, sVCAM-1, and PAI-1 levels with CSF routine parameters were detected. In addition, the levels of the adhesion molecules were not significantly correlated with previous suicide attempts and the number of earlier inpatient stays.

## 4. Discussion

The results of this study revealed significantly elevated sICAM-1 and sVCAM-1 levels in patients suffering from schizophrenia spectrum disorders compared with patients with depressive disorders. Oriented to established CSF reference values (using ELISA-technique) of sICAM according to which CSF values < 300 pg/mL must be assumed in healthy controls, the values in depressed patients (mean: 466.205 pg/mL) have been found to be already slightly increased. Those in schizophreniform disorders were clearly elevated and on average four times above the established reference value (mean: 1196 pg/mL) (for reference values, see: https://07525720-0688-4380-840d-0a4af942fef7.filesusr.com/ugd/92c932_454e4d6908d94f64b3623b621179eade.pdf; accessed on 12 June 2021). An upregulation of these signaling molecules in the schizophrenia spectrum disorder cohort may first be indicative of neuroinflammatory processes followed by a proinflammatory immune response [3,26]. Second, the overexpression of the adhesion molecules may be related to an impairment of the BBB/BCSFB [3,21]. Accordingly, the sVCAM-1 levels correlated with the AQ (which is considered the gold standard in estimating the integrity of the BBB/BCSFB) in patients with schizophrenia spectrum disorders [6,14,15,27,28].

### 4.1. Integration of Our Findings into the Context of the Current Research

Adhesion molecules are relevant for the maintenance and formation of the neuronal structure [29], and deficits in the neuronal structure have been consistently associated with the pathophysiology of schizophrenia [30,31,32]. Increased sICAM-1 levels have been observed in multiple inflammatory and cell-mediated autoimmune disorders [32]. The current findings of increased CSF levels of this molecule are consistent with the reported significant elevation of plasma sICAM-1 levels in patients with schizophrenia spectrum disorder [5,23]. Stefanovic et al. (2016) discerned increased sICAM-1 levels in patients at a late stage of the disease, whereas no difference between healthy controls and patients with schizophrenia spectrum disorder was found in the early disease stages [5]. By contrast, decreased peripheral levels of sICAM-1 and sVCAM-1 have been reported in another cohort of patients with schizophrenia spectrum disorders [22]. In the explanatory approach, these contradictory findings may be explained in light of dysfunctional neuroendocrine immune communication and a reduced immune response during the acute onset of schizophrenia. On the other hand, overexpression could be an indication of an immune activation during a prolonged course of the disease [3,4]. Consistent with this view, 68% (27 out of 40) of the patients with schizophrenia in the present cohort suffered from a recurrent/chronic course of the disease. However, we could not detect significant differences that distinguished patients with the first episode from those with the recurrent/chronic stage. In the first uncontrolled CSF study on cell adhesion molecules in schizophrenia, a significant correlation was found between sICAM-1 levels and AQs [21]. This finding could not be replicated in our data. However, a significant positive correlation between sVCAM-1 levels and AQs was discerned (see Figure 1).

### 4.2. Pathophysiological and Clinical Considerations

An increase in circulating proinflammatory cytokines was determined in the context of multiple psychiatric disorders [4,23,33]. Different inflammatory mediators (e.g., TNFα, IL-1β, and IFNγ) induce the expression levels of ICAM-1 and VCAM-1 [4,9,10,18]. ICAM-1 (CD54) is a transmembrane glycoprotein approximately 100 kDa in size. It belongs to the immunoglobulin supergene family and consists of five tandem immunoglobulin-like domains [3,24,26,33]. In the CNS, ICAM-1 is expressed most notably in microglial cells, astrocytes, and endothelial cells in the white and grey matter [3,26]. The ligations of ICAM-1 to the lymphocyte function-associated molecule 1 on the surface of endothelial cells and to the macrophage-associated antigen-1 receptors on leucocytes contribute to immune cell infiltration during an inflammatory response [23,24,33]. ICAM-1 enables the trans-endothelial migration of leukocytes to the site of inflammation and plays an important role in the interaction between antigen-presenting cells and T cells in lymphocyte activation and in numerous cellular immune responses [3,23,26,32,33]. Arising from alternative splicing and/or proteolytic cleavage of membrane-bound ICAM-1 messenger RNA, a circulating soluble form of ICAM-1 (sICAM-1) consisting of the complete extracellular domain can be found in the serum and CSF [24,26,33]. The sICAM-1 and its membrane-bound form exert similar functions [3]. The elevated levels of sICAM-1 in CSF—as demonstrated in the current study—or in serum may therefore be indicative of the upregulated state of the membrane-bound ICAM-1 in the brain [3]. VCAM-1 (CD106) is a 90 kDa glycoprotein predominantly expressed in endothelial cells [18]. VCAM-1 regulates the pathway involved in leukocyte recruitment and transendothelial migration during inflammation via the interaction of its domain 1 (and/or 4) with α4β1 integrin [18]. In most cell types, the expression of leucocyte adhesion molecules, such as ICAM-1 and VCAM-1, is low under non-inflammatory conditions. In contrast, a state of overexpression was described in many pathological states, especially during chronic inflammatory processes [3,6,18,26]. Given that ICAM-1 is widely expressed in tissues, ICAM-1 levels may indicate a general level of inflammation [32]. By contrast, VCAM-1 seems to indicate the conditions of the cerebral endothelium and the dendritic cells more precisely and thus could be used to assess endothelial dysfunction [32]. Correlations were observed between elevated levels of ICAM-1 and the progression and severity of cancer, cardiovascular disease, and autoimmune disorders [3,33]. They were also observed between VCAM-1 and the progression of various immunological disorders, including rheumatoid arthritis and cancer [18]. In patients with schizophrenia spectrum disorders, the current study showed evidence of upregulated ICAM-1 and VCAM-1 levels, which may partially reflect the occurrence of leukocyte transendothelial recruitment and adhesion [3]. The overexpression of ICAM-1 and VCAM-1 near the endothelial layer of the vessel wall impairs vascular endothelial mitochondrial oxidative metabolism and directly destabilizes endothelial tight junctions [4,9,10,18]. These processes increase BBB/BCSFB permeability and allow the inappropriate migration of pro-inflammatory molecules into the brain parenchyma, enabling interactions between the innate and peripheral adaptive immune systems in the brain [9,16,18,34]. These theoretical considerations are supported by the correlation found between sVCAM-1 levels and AQs in this study. In addition, it was demonstrated earlier that ICAM-1 and VCAM-1 can be elevated without an “open BBB/BCSFB”. In a review by Varatharaj and Galea (2017), disruptive and nondisruptive changes in the BBB were compared. The fact that there was no severe barrier disruption (with elevated AQs) across the entire present cohort, but already high sICAM-1 and sVCAM-1 levels, could indicate that nondisruptive changes are underlying the pathological processes here. Therefore, the tight junctions would not be affected, but the endothelia would still let immune cells pass or secrete cytokines/chemokines. Thus, from a clinical perspective, sICAM-1 and sVCAM-1 could provide further information about the BBB/BCSFB function in addition to established CSF parameters such as AQ.

### 4.3. Limitations

A limitation of the present study is its lack of a healthy control group, especially with regard to CSF measurements. It is difficult to ethically justify lumbar punctures in a large group of healthy volunteers. Previously, we used a control group of patients with pseudotumor cerebri (e.g., [25,35]). In the current study, this approach was initially considered; unfortunately, we were unable to recruit a matched control group with an adequate sample size. However, we were able to use a clinical control group of patients with depressive disorders and established reference values. Patients with schizophrenia were routinely offered a lumbar puncture. For patients with depression, lumbar punctures were performed only in selected cases. These patients were not screened routinely, and there probably is a selection bias towards severely depressed patients. In addition, the multiplexing immunoassay used was originally not established for CSF measurements, and its use may have led to methodological inaccuracies and difficulties. However, most other methodological approaches have so far only been established for blood. Because CSF analysis was performed as part of a routine clinical diagnostic work-up, the processes involved in sample processing were not completely standardized. The samples first underwent routine testing before being frozen at −80 °C. The procedure was the same in both groups. In future studies, samples should be processed directly according to established and pre-defined standard operating procedures. The influence of other possible contributing factors, such as psychotropic medication, substance abuse, or medical conditions, including multiple vascular risk factors [3,4,36], remains unclear and needs to be considered. An influence of psychotropic drugs on our results could not be detected in a secondary analysis. In addition, we were unable to examine the serum samples of the patients. This would have been helpful for the overall interpretation and comparison with the preliminary studies, which mostly only examined serum material. Finally, it is important to keep in mind that the overexpression of ICAM-1 is observed in a wide range of diseases and inflammation, even in depressive disorders. Therefore, the present findings in patients with schizophrenia spectrum disorder probably do not reflect disease-specific processes [3]. Due to the limitations mentioned above, the present results should be considered preliminary and warrant the replication in future studies.

## 5. Conclusions

The schizophrenia spectrum disorder pathophysiology may involve an altered immune response and disturbed communication between the CNS and the immune system due to an impaired BBB/BCSFB. The present results indicate that the circulating immune signaling molecules sICAM-1 and sVCAM-1 might play a relevant role in this context. Further translational, prospective, and controlled studies in this novel psychoneuroimmunological field of research are needed.

## Figures and Tables

**Table 1:** Overview of findings measuring levels of sICAM-1 and sVCAM-1 in patients with schizophrenia-spectrum disorders (reviewed by Müller, 2019 [3]).

Sample Material	Method	Research Group	Schizophrenia-Spectrum Disorder Group	Control Group	Results
CSF	ELISA	Schwarz et al., 1998 [21]	*n* = 40	Ø	Significant association of sICAM-1 and BCSFB
CSF	ELISA	Schwarz et al., 2000 [22]	*n* = 18	Ø	Significant positive correlation of sICAM-1 with negative symptomatology and disease duration
Cortex tissue	PCR	Cai et al., 2018 [23]	*n* = 37	*n* = 37	↑ expression of ICAM-1 mRNA
Plasma	Multiplexing immunoassay (Luminex^®^)	Cai et al., 2018 [23]	*n* = 78	*n* = 73	↑ levels of sICAM-1
Plasma	Multiplexing immunoassay (Meso Scale Discovery MULTI-SPOT^®^)	Nguyen et al., 2018 [4]	*n* = 134	*n* = 113	↑ levels of ‘vascular endothelial index’ including VEGF, sICAM-1, sVCAM-1
Serum	ELISA	Schwarz et al., 2000 [22]	*n* = 72	*n* = 38	↓ levels of sICAM-1 and increase of sICAM-1 during treatment
Serum	ELISA	Kronig et al., 2005 [24]	*n* = 70	*n* = 128	↓ levels of sICAM-1 and relationship to ICAM-1 G214A polymorphism
Serum	ELISA	Stefanović et al., 2016 [5]	*n* = 80	*n* = 80	= levels of sICAM-1 in early-stage, levels of sICAM-1 in late-stage and associations with severity and disease duration

Abbreviations: CSF, cerebrospinal fluid; ELISA, enzyme-linked immunosorbent assay; sICAM-1, soluble intercellular adhesion molecule 1; BCSFB, blood–CSF-barrier; PCR, polymerase chain reaction; mRNA, messenger RNA; VEGF, vascular endothelial growth factor; sVCAM-1, vascular cell adhesion molecule 1; ↑, higher; =, normal; ↓, lower; Ø, none.

**Table 2:** Clinical data of patients with schizophrenia-spectrum disorder and depressive disorder.

	Schizophrenia-Spectrum Disorder (*n* = 40)	Depressive Disorder (*n* = 39)
**Sex**	16 M: 24 F	14 M: 25 F
**Age** (Mean ± SD, range)	33.63 ± 13.38	32.54 ± 7.65
	(18–65 years)	(18–44 years)
**Clinical Syndrome and Characteristics**		
Severe depressive episode		39 (100%)
With psychotic symptoms		7 (18%)
Without psychotic symptoms		32 (82%)
Schizophrenia spectrum disorder	40 (100%)	
Paranoid-hallucinatory	25 (63%)	
Hebephrenic	1 (3%)	
Catatonic	1 (3%)	
Delusional disorders	1 (3%)	
Schizoaffective	11 (28%)	
- Depressive	6 (15%)	
- Manic	3 (8%)	
- Mixed	2 (5%)	
Acute polymorphic psychotic	1 (3%)	
**Course of Disease**		
Recurrent/chronic	27 (68%)	27 (69%)
First episode	13 (33%)	12 (31%)
**Neurologic Comorbidity**		
Seizures/Attacks	2 (5%)	0 (0%)
Traumatic	3 (8%)	0 (0%)
Polyneuropathy	0 (0%)	0 (0%)
Migraine/Headache	1 (3%)	1 (3%)
Overall	6 (15%)	1 (3%)
**Psychotropic Medication at the Time of Sampling**		
SSRI	4 (10%)	9 (23%)
SSNRI	1 (3%)	21 (54%)
Tricyclic antidepressants	0 (0%)	8 (21%)
Bupropion	0 (0%)	4 (10%)
Mirtazapine	1 (3%)	6 (15%)
Typical neuroleptics	9 (23%)	4 (10%)
Atypical neuroleptics	40 (100%)	21 (54%)
Lithium	7 (18%)	9 (23%)
Anticonvulsant	7 (18%)	1 (3%)
Benzodiazepine	9 (23%)	3 (8%)
Unmedicated	0 (0%)	2 (5%)

Abbreviations: CSF = cerebrospinal fluid, MRI = magnetic resonance imaging, EEG = electroencephalography, F = female, M = male, SD = standard deviation, SSRI = selective serotonin reuptake inhibitor, SSNRI = selective serotonin/noradrenaline reuptake inhibitor.

**Table 3:** Demographic data.

	Schizophrenia-Spectrum Disorder (*n* = 40)	Depressive Disorder (*n* = 39)	Statistics
**Marital status**			n.s.
Single	30 (77%)	31 (79%)	
Married	6 (15%)	6 (15%)	
Divorced	1 (3%)	2 (5%)	
Widowed	1 (3%)	0 (0%)	
Unknown	2 (5%)	0 (0%)	
**Level of education**			***p* = 0.013**
Low	11 (28%)	2 (5%)	
Middle	7 (18%)	8 (21%)	
High	19 (48%)	28 (72%)	
Unknown	3 (8%)	1 (3%)	
**Work situation**			n.s.
Unemployed	7 (18%)	6 (15%)	
Working	13 (33%)	20 (51%)	
In training	11 (28%)	11 (28%)	
Retired	6 (15%)	1 (3%)	
Housewife/-man	2 (5%)	1 (3%)	
Unknown	1 (3%)	0 (0%)	
**Housing situation**			n.s.
Alone	13 (33%)	18 (47%)	
With partner/family	11 (28%)	10 (26%)	
With parents/guardian	12 (30%)	10 (26%)	
Other	4 (10%)	0 (0%)	
Unknown	0 (0%)	1 (3%)	
**Suicide attempts**			n.s.
None	28 (70%)	34 (87%)	
One	2 (5%)	2 (5%)	
Two	4 (10%)	2 (5%)	
Three	1 (3%)	0 (0%)	
Four	1 (3%)	0 (0%)	
Five	1 (3%)	0 (0%)	
Six	0 (0%)	1 (3%)	
Unclear	3 (8%)	0 (0%)	
**Number of earlier inpatient treatments**			n.s.
None	12 (30%)	15 (38%)	
One	6 (15%)	12 (31%)	
Two	3 (8%)	6 (15%)	
Three	3 (8%)	2 (5%)	
Four	2 (5%)	1 (3%)	
Five	5 (13%)	2 (5%)	
>Five	7 (18%)	1 (3%)	
Unclear	2 (5%)	0 (0%)	

Abbreviation: n.s. = not significant.

**Table 4:** Cell adhesion molecule levels in the cerebrospinal fluid.

	Schizophrenia-Spectrum Disorder (*n* = 40)	Depressive Disorder (*n* = 39)	Statistics
PAI-1 (pg/mL) (Mean ± SD)	72.006 ± 46.810	30.756 ± 23.397	z = −4.266 *p* < 0.001
sICAM-1 (pg/mL) (Mean ± SD)	1196.252 ± 768.714	466.205 ± 277.053	z = −5.753 *p* < 0.001
sVCAM-1 (pg/mL) (Mean ± SD)	456.197 ± 155.549	234.195 ± 151.553	z = −5.354 *p* < 0.001

Abbreviations: PAI-1 = plasminogen activator inhibitor 1, SD = standard deviation, s-ICAM-1 = soluble intercellular adhesion molecule-1, sVCAM-1 = soluble vascular cell adhesion molecule-1.

**Table 5:** Findings in cerebrospinal fluid routine diagnostics.

	Reference	Schizophrenia-Spectrum Disorder (*n* = 40)	Depressive Disorder (*n* = 39)	Statistics
**WBC counts** (Mean ± SD)	in/µL	1.85 ± 1.46	1.82 ± 1.23	Z = −0.189 *p* = 0.850
**Number of increased WBC counts**	<5/µL	↑: 3 (8%)	↑: 2 (5%)	*p* = 1.00
**Protein concentration** (Mean ± SD)	in mg/L	406.45 ± 196.15	418.87 ± 153.68	z = −1.098 *p* = 0.272
**Number of increased protein concentration**	<450 mg/L	↑: 12 (30%)	↑: 14 (36%)	Chi^2^ = 0.311 *p* = 0.577
**Albumin quotient** (Mean ± SD)		5.02 ± 2.29	5.12 ± 2.05	z = −0.608 *p* = 0.543
**Number of increased albumin quotients**	<40 y.: <6.5 × 10^−3^ 40–60 y.: <8 × 10^−3^ >60 y.: <9.3 × 10^−3^	↑: 6 (15%)	↑: 8 (21%)	Chi^2^ = 0.412 *p* = 0.521
**IgG-Index** (Mean ± SD)	in mg/L	0.49 ± 0.04	0.49 ± 0.09	z = −0.005 *p* = 0.996
**Number of increased IgG indices**	<0.7 mg/L	↑: 0 (0%)	↑: 1 (3%)	*p* = 0.494
**OCBs in CSF**	negative	1* (3%)	2 (5%)	Chi^2^ = 0.556 *p* = 0.346

Abbreviations: WBC = white blood cell, SD = standard deviation, y. = years, IgG = immunoglobulin G, CSF = cerebrospinal fluid, OCBs = oligoclonal bands. * Two findings were borderline positive: A first patient had some weak identical bands in CSF and serum, a second patient had an isolated OCB in the CSF.

## Data Availability

All relevant results are presented in the manuscript.
